# Asthma during Pregnancy in a Population-Based Study - Pregnancy Complications and Adverse Perinatal Outcomes

**DOI:** 10.1371/journal.pone.0104755

**Published:** 2014-08-20

**Authors:** Gustaf Rejnö, Cecilia Lundholm, Tong Gong, Kjell Larsson, Sissel Saltvedt, Catarina Almqvist

**Affiliations:** 1 Department of Medical Epidemiology and Biostatistics, Karolinska Institutet, Stockholm, Sweden; 2 Obstetrics and Gynaecology Unit, Södersjukhuset, Stockholm, Sweden; 3 Institute of Environmental Medicine, Karolinska Institutet, Stockholm, Sweden; 4 Department of Clinical Science and Education, Södersjukhuset, Karolinska Institutet, Stockholm, Sweden; 5 Obstetrics and Gynaecology Unit, Karolinska University Hospital, Stockholm, Sweden; 6 Astrid Lindgren Children's Hospital, Lung and Allergy Unit, Karolinska University Hospital, Stockholm, Sweden; Oslo University Hospital, Ullevål, Norway

## Abstract

**Background:**

Asthma is one of the most common chronic diseases, and prevalence, severity and medication may have an effect on pregnancy. We examined maternal asthma, asthma severity and control in relation to pregnancy complications, labour characteristics and perinatal outcomes.

**Methods:**

We retrieved data on all singleton births from July 1, 2006 to December 31, 2009, and prescribed drugs and physician-diagnosed asthma on the same women from multiple Swedish registers. The associations were estimated with logistic regression.

**Results:**

In total, 266 045 women gave birth to 284 214 singletons during the study period. Maternal asthma was noted in 26 586 (9.4%) pregnancies. There was an association between maternal asthma and increased risks of pregnancy complications including preeclampsia or eclampsia (adjusted OR 1.15; 95% CI 1.06–1.24) and premature contractions (adj OR 1.52; 95% CI 1.29–1.80). There was also a significant association between maternal asthma and emergency caesarean section (adj OR 1.29; 95% CI 1.23–1.34), low birth weight, and small for gestational age (adj OR 1.23; 95% CI 1.13–1.33). The risk of adverse outcomes such as low birth weight increased with increasing asthma severity. For women with uncontrolled compared to those with controlled asthma the results for adverse outcomes were inconsistent displaying both increased and decreased OR for some outcomes.

**Conclusion:**

Maternal asthma is associated with a number of serious pregnancy complications and adverse perinatal outcomes. Some complications are even more likely with increased asthma severity. With greater awareness and proper management, outcomes would most likely improve.

## Introduction

Asthma is one of the most common chronic diseases with reported prevalence between 3.7 and 8.4% [1], [2]. Asthma symptoms may increase during pregnancy which can be attributable either to worsening of asthma due to the pregnancy itself or inadequate medication [3], [4]. Maternal asthma has been associated with an increased risk for pregnancy complications such as preeclampsia, gestational diabetes, premature contractions and premature ruptures of membranes [5]–[8]. Maternal asthma has also been associated with an increased prevalence of labour characteristics including caesarean section and placental abruption [9]–[12], or perinatal outcomes such as low birth weight, prematurity and small for gestational age (SGA) [6], [12]–[15] but studies are also non-consistent [2], [7], [16].

According to GINA (Global Initiative for Asthma) guidelines [17], asthma can be classified into mild, moderate and severe and symptoms should be treated with medication in a step-wise approach to obtain asthma control. Mild and moderate well-controlled asthma is generally associated with uncomplicated pregnancy and delivery [8]. More importantly, severe asthma and poor asthma control seem to increase the risk of preterm delivery, low birth weight and small for gestational age (SGA) [18]. Yet, not all studies have reported increased risks of perinatal complications with severe asthma and poor control. A recent meta-analysis suggested that this discrepancy might be caused by variation in study size and that participation in smaller prospective studies is associated with better disease control and therefore better outcomes [19]. It is of great importance that midwives and obstetricians identify pregnant women at risk of adverse outcomes due to asthma. Thus, there is a call for larger studies to assess the effect of maternal asthma, severity and control on pregnancy complications, labour and perinatal outcomes.

Findings on the effect of maternal asthma on pregnancy and delivery outcomes in population-based cohorts compiled from registers offers many advantages including powerful assessment of the association between asthma in pregnant women and pregnancy complications or adverse outcomes, with control for important confounders and the effect of medication usage. By using information on asthma diagnosis from different sources and prescribed drugs as a proxy for disease it is possible to obtain objective measures of exposure. Indices developed to assess the severity and control of asthma can be used in epidemiological studies [20], [21].

The principal aim of this study was to assess possible associations between maternal asthma, pregnancy and labour characteristics and adverse perinatal outcomes. We also aimed to assess the effect of asthma severity and control on pregnancy complications and perinatal outcomes.

## Materials and Methods

### Study design and population

The Swedish National Board of Health and Welfare holds a number of registers covering health information. The universal use of the Personal Identity Number (PIN), a unique identifier for each resident, enables unambiguous linkage to these registers and those held by Statistics Sweden [22]. We conducted a population-based cohort study using the four Swedish national registers held by the Swedish National Board of Health and Welfare and Statistics Sweden.

All women in Sweden who started their pregnancy after July 1 2006, bore singletons and gave birth at the latest 31 December 2009 were identified through the Medical Birth Register, which includes data on pregnancy and perinatal characteristics for approximately 99% of all births, after pregnancy week 22, in Sweden since 1973 [23]. The cohort was linked to the Prescribed Drug Register which contains all prescribed drugs dispensed at pharmacies in Sweden since July 1, 2005 [24], the National Patient Register which covers all in-patient care in Sweden from 1987 and 75% of all outpatient visits since 2001. Information on socioeconomic status including cohabitation and level of education was retrieved from the Longitudinal integration database for health insurance and labour market studies (LISA by Swedish acronym), held by Statistics Sweden.

We included only singletons since multiple pregnancies exhibit many different characteristics compared to singletons.

### Variables

#### Maternal characteristics

Maternal age and body mass index (BMI in kg/m^2^), calculated from height and weight, were retrieved from the Medical Birth Register. Other background characteristics from the same source were parity, smoking, country of birth, cohabitation. Variables on maternal age and parity were recorded at delivery whereas maternal smoking habits, height and weight were registered at the first visit to the antenatal-care clinic in week 8–12. From LISA we collected information on marital status and highest level of maternal education at start of pregnancy.

#### Maternal asthma – diagnosis and medication

We used multiple registers to identify individuals with asthma in the study population. In the Medical Birth Register, a tick-box for self-reported asthma/lung disease ever is indicated by the midwife at antenatal care admission in early pregnancy. From the National Patient Register we obtained information on specialist care visits with a recently validated recorded diagnosis of asthma (ICD-10 codes J45, J46) twelve months before and during pregnancy [25]. Information on dispensed asthma medication before and during pregnancy was obtained from ATC-codes (Anatomical Therapeutic Chemical Classification) R03 (inhaled corticosteroids (ICS), short-acting β2-agonists (SABA), long-acting β2-agonists (LABA), leukotriene receptor antagonist (LTRA), anticholinergic inhalers, sodium chromoglycate, theophylline and omalizumab) and H02 (oral corticosteroids) in the Prescribed Drug Register. Having had asthma medication dispensed at least twice during the year before pregnancy until birth date was used as a proxy for an asthma diagnosis. We excluded oral β2-agonists (ATC code R03CC) since this is not only prescribed for asthma but sometimes for premature contractions. Our main exposure was asthma recorded in any of the three registers the Medical Birth Register, the National Patient Register or the Prescribed Drug Register.

In order to assess usage of asthma medication during different pregnancy trimesters, midwife reports were extracted from the Medical Birth Register along with gestational week of medication start and discontinuation.

We defined asthma severity and control based on medication use in the Prescribed Drug Register twelve months prior to pregnancy modified from Firoozi et al [21]. Average daily dose of ICS, SABA and two prescriptions per year of LABA, theophylline, and LTRA, along with asthma diagnoses in the National Patient Register inpatient part [26] and/or filled prescription of an oral corticosteroid over 12 months were used to categorize the mothers into mild or moderate/severe asthma that could be either controlled or uncontrolled, Appendix S1. The severity index was modified by changing the number of filled prescriptions for LABA, theophylline and LTRA from six to two over a 12 months period for adhering more to local prescription patterns.

#### Outcomes

All the outcome variables were collected from the Medical Birth Register. Based on pregnancy and delivery ICD-10 codes recorded we could collect information on preeclampsia/eclampsia (ICD-10: O14–15), gestational diabetes (ICD-10: O24), haemorrhage during pregnancy (ICD-10: O46), premature contractions (ICD-10: O47) premature rupture of membranes, PROM (ICD-10: O42) and placental abruption (ICD-10: O45).

We also included the following labour information: dystocia during labour, induced/spontaneous onset of labour, caesarean section (elective or emergency caesarean section prior to onset of labour, or emergency CS after onset of labour) and vaginal instrumental delivery (ICD-10-codes O62, O66.5, O81, O82 for forceps or vacuum extraction). Based on these, delivery mode was categorized as: 1) Vaginal, non-instrumental delivery, 2) Elective caesarean (before start of labour), 3) Vaginal instrumental delivery and 4) Emergency caesarean section (prior to or after start of labour).

For birth outcomes and post-partum characteristics we used data on birth weight, gestational age, small (SGA)/large (LGA) for gestational age (birth weight >2 standard deviations below or above reference curve for children of similar gestational age) [27], Apgar at 5 minutes, information on haemorrhage after delivery (ICD-10: O72), and asphyxia/hypoxia (ICD-10: P20–P21).

### Statistical analysis

We used logistic and multinomial logistic regression analysis to estimate odds ratios (OR) as the measure of association, with 95% confidence intervals (CI) for the outcomes in relation to asthma in any of the three registers as well as asthma medication in the Medical Birth Register, asthma severity and asthma control the year before pregnancy. We estimated both the crude ORs and adjusted for maternal age, BMI, parity, smoking at start of pregnancy, cohabitation/marital status, education and country of birth. To account for the clustering of observations within women with multiple deliveries the sandwich estimator for the standard errors was used. The Holm-Bonferroni method was used to evaluate what significant results from the primary analyses (i.e. adjusted analyses of maternal asthma yes/no and each of the outcome variables) there would be if applying an overall 5% significance level for those tests, To test for effect modification of asthma control by disease severity we included interaction terms in the models and tested for significance with likelihood-ratio test. STATA 12.1 was used for the analyses.

Permission for this study was obtained from the Regional Ethical Review board, “Regionala etikprövningsnämnden – EPN”, in Stockholm, Sweden. In accordance with their decision, we did not obtain informed consent from participants involved in the study. All data were made anonymous prior to analyses.

## Results

In this register-based cohort study, 284 214 pregnancies were completed in 266 045 women during the study period. The majority of women gave birth to only one child, but some women had two (18 003 women) or three (83 women) pregnancies within the period. Asthma was recorded for 26 586 (9.4%) of all pregnancies.

Maternal characteristics in relation to maternal asthma in any of the three registers are shown in Table 1. Compared to women without asthma, those with asthma were slightly younger (16.0% versus 14.6% below 25 years of age), had higher BMI, and were more often primiparous and smokers, more often born in Sweden, less often living with the baby's father and had lower education.

**Table 1:** Background characteristics of study population of 284 214 pregnancies by asthma status.

	Asthma* No	Asthma* Yes
	n = 257 628	n = 26 586
	n(%)	n(%)
Maternal Characteristics		
***Age***		
*≤19*	4312 (1.7)	519 (2.0)
*20*–*24*	33 316 (12.9)	3721 (14.0)
*25*–*29*	73 744 (28.6)	7626 (28.7)
*30*–*34*	89 948 (34.9)	8973 (33.8)
*>34*	56 308 (21.9)	5747 (21.6)
***BMI***		
*<18,5*	5763 (2.2)	518 (1.9)
*18,5*–*24,9*	145 226 (56.4)	13 288 (50.0)
*25*–*29,9*	57 913 (22.5)	6740 (25.4)
*≥30*	26 471 (10.3)	4222 (15.9)
*Missing*	22 255 (8.6)	1818 (6.8)
***Parity***		
*1*	149 334 (58.0)	16 065 (60.4)
*2*–*3*	94 090 (36.5)	8937 (33.6)
*≥4*	14 204 (5.5)	1584 (6.0)
***Sex of offspring***		
*Girl*	124 881 (48.5)	12 966 (48.8)
*Boy*	132 747 (51.5)	13 620 (51.2)
***Cigarettes smoked/day***		
*0*	229 737 (89.2)	23 452 (88.2)
*1*–*9*	12 774 (5.0)	1821 (6.8)
*>9*	3684 (1.4)	620 (2.3)
*Missing*	11 433 (4.4)	693 (2.6)
***Country of birth***		
*Sweden*	198 585 (77.1)	22 945 (86.3)
*Denmark, Norway, Finland, Iceland*	4051 (1.6)	411 (1.5)
*Other countries*	54 845 (21.3)	3221 (12.1)
*Missing*	147 (0.1)	9 (0.0)
***Cohabitation/marital status***		
*Living with baby*'*s father, married*	105 247 (40.9)	9788 (36.8)
*Living with baby*'*s father, unmarried*	124 405 (48.3)	14 107 (53.1)
*Not living with baby*'*s father*	13 673 (5.3)	1840 (6.9)
*Missing*	14 303 (5.6)	851 (3.2)
***Mother's education level***		
*≤9 years*	27 491 (10.7)	3141 (11.8)
*10*–*12 years*	96 841 (37.6)	10 870 (40.9)
*13*–*14 years*	30 287 (11.8)	3124 (11.8)
*≥15 years*	92 756 (36.0)	9038 (34.0)
*Missing*	10 253 (4.0)	413 (1.6)

*Asthma recorded in the Swedish Medical Birth Register, asthma diagnosis in the Swedish National Patient Register and/or asthma medication suspended at least twice according to the Swedish Prescribed Drug Register.

Maternal asthma was associated with an increased risk of almost all pregnancy complications, Table 2. For example, there was a 15% increased odds of preeclampsia or eclampsia (95% CI 1.06–1.24) in the group with maternal asthma and an 34% increased odds of haemorrhage during pregnancy (95% CI 1.12–1.60) and premature contractions which were increased by 52% (95% CI 1.29–1.80). There were also increased odds for adverse birth outcomes, including low birth weight and low gestational age. After adjusting for multiple testing most statistically significant associations remained except for lowest birth weight category and LGA. Subanalyses using asthma medication or diagnosis in the three different registers separately showed similar estimates, data not tabulated.

**Table 2:** Associations between maternal asthma and pregnancy characteristics, labour characteristics and perinatal outcomes in a cohort of 284 214 pregnancies, reported as unadjusted and adjusted OR with 95% CI, all estimated by multinomial logistic regression.

	Number of pregnancies	Asthma§	Unadjusted OR (95% CI)	Adjusted† OR (95% CI)	P-values adjusted
	N (%)	n(%)			
**Pregnancy Characteristics**					
***Preeclampsia or eclampsia***	7797 (2.7)	915 (3.4)	1.30 (1.21–1.39)	1.15 (1.06–1.24)	<0.001*
***Gestational diabetes***	4804 (1.7)	534 (2.0)	1.22 (1.10–1.34)	1.09 (0.99–1.20)	0.066
***Haemorrhage during pregnancy***	1341 (0.5)	151 (0.6)	1.23 (1.03–1.48)	1.34 (1.12–1.60)	0.001*
***Premature contractions***	1497 (0.5)	211 (0.8)	1.59 (1.37–1.85)	1.52 (1.29–1.80)	<0.001*
***Premature rupture of membranes***	4630 (1.6)	544 (2.0)	1.30 (1.19–1.41)	1.30 (1.18–1.43)	<0.001*
***Placental abruption***	1053 (0.4)	136 (0.5)	1.44 (1.21–1.71)	1.44 (1.18–1.75)	<0.001*
**Labour Characteristics**					
***Labour dystocia***	29 523 (10.4)	3040 (11.4)	1.13 (1.08–1.17)	1.10 (1.06–1.15)	<0.001*
***Mode of delivery***					
*Vaginal non-instrumental delivery*	213 687 (75.2)	18 916 (71.2)	Ref.	Ref.	
*Elective CS (before start of labour)*	19 396 (6.8)	2191 (8.2)	1.31 (1.25–1.38)	1.29 (1.22–1.36)	<0.001*
*Vaginal instrumental delivery*	21 792 (7.7)	2105 (7.9)	1.10 (1.05–1.15)	1.11 (1.06–1.17)	<0.001*
*Emergency CS prior to or after start of labour*	27 632 (9.7)	3177 (11.9)	1.34 (1.29–1.39)	1.29 (1.23–1.34)	<0.001*
*Missing*	1707 (0.6)	197 (0.7)			
***Haemorrhage after delivery***	14 765 (5.2)	1413 (5.3)	1.03 (0.97–1.08)	1.03 (0.97–1.09)	0.358
**Birth outcome and post-partum**					
***Birth weight, grams***					
*≤1999*	3809 (1.3)	370 (1.4)	1.08 (0.96–1.20)	1.15 (1.02–1.29)	0.034
*2000*–*2499*	5811 (2.0)	637 (2.4)	1.23 (1.13–1.34)	1.32 (1.20–1.45)	<0.001*
*2500*–*2999*	30 118 (10.6)	3109 (11.7)	1.15 (1.10–1.20)	1.29 (1.23–1.34)	<0.001*
*3000*–*3499*	92 210 (32.4)	8615 (32.4)	1.03 (1.00–1.06)	1.12 (1.09–1.16)	<0.001*
*≥3500*	151 827 (53.4)	13 810 (51.9)	Ref.	Ref.	
*Missing*	439 (0.2)	45 (0.2)			
***Gestational age, weeks***					
*≤31*	2398 (0.8)	221 (0.8)	1.03 (0.89–1.18)	1.05 (0.90–1.22)	0.671
*32*–*34*	3489 (1.2)	371 (1.4)	1.20 (1.08–1.35)	1.19 (1.06–1.33)	0.002*
*35*–*36*	8757 (3.1)	1030 (3.9)	1.35 (1.26–1.44)	1.30 (1.21–1.39)	<0.001*
*37*–*38*	54 084 (19.0)	5601 (21.1)	1.17 (1.13–1.21)	1.18 (1.13–1.22)	<0.001*
*39*–*40*	145 298 (51.1)	13 061 (49.1)	Ref.	Ref.	
*≥41*	69 993 (24.6)	6293 (23.7)	1.00 (0.97–1.03)	0.97 (0.94–1.01)	0.165
*Missing*	195 (0.1)	9 (0.0)			
***Small for gestational age***					
*Yes*	6722 (2.4)	714 (2.7)	1.16 (1.07–1.25)	1.23 (1.13–1.33)	<0.001*
*Missing*	625 (0.2)	54 (0.2)			
***Large for gestational age***					
*Yes*	9639 (3.4)	977 (3.7)	1.10 (1.03–1.17)	0.93 (0.86–0.99)	0.035
*Missing*	625 (0.2)	54 (0.2)			
***Apgar at 5 minutes <7***	3322 (1.3)	384 (1.4)	1.10 (0.99–1.23)	1.08 (0.96–1.20)	0.198
***Asphyxia or hypoxia***	2873 (1.0)	297 (1.1)	1.12 (0.99–1.26)	1.07 (0.94–1.22)	0.291

In the unadjusted analyses n = 26 586 pregnancies were included in the asthma group. In the adjusted analyses n = 24 203 pregnancies were included in the asthma group.

§ Asthma recorded in the Swedish Medical Birth Register, asthma diagnosis in the Swedish National Patient Register and/or asthma medication suspended at least twice according to the Swedish Prescribed Drug Register.

† Adjusted for age, BMI, parity, smoking at antenatal care admission, country of birth, cohabitation/marital status and level of education.

*Significant after having adjusted for multiple hypotheses testing using the Holm-Bonferroni method.

**Figure 1:**
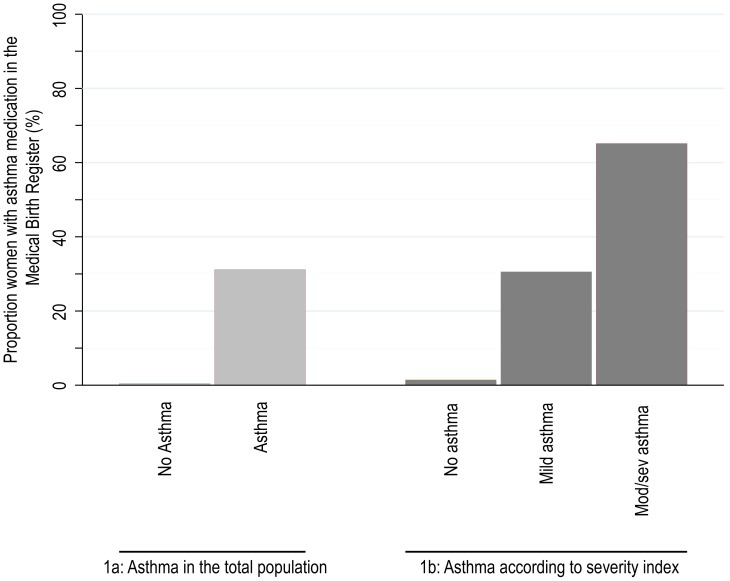
Asthma medication in the Medical Birth Register. a) Proportion of women with asthma medication recorded in the Medical Birth Register if asthma reported in the Medical Birth Register, National Patient Register or the Prescribed Drug Register. b) Proportion of women with asthma medication in the Medical Birth Register among those with no, mild and moderate/severe asthma according to the modified Firoozi severity index.

Among women with asthma in any of the health registers, 31% also had asthma medication recorded in the Medical Birth Register during pregnancy and only 0.4% had asthma medication during pregnancy if no asthma was noted in the registers, Figure 1a. According to the severity index, 13 034 (4.6% of the population) had asthma the year prior to conception. Of those, 32% were classified as mild controlled asthma, 37% as mild uncontrolled, 12% as moderate controlled, 15% as moderate uncontrolled, 0.6% as severe controlled and 4% as severe uncontrolled asthma. Among the 8962 women with mild asthma according to the severity index, 31% had asthma medication noted in the Medical Birth Register and 65% of the 4072 women with moderate or severe asthma, Figure 1b.

**Figure 2:**
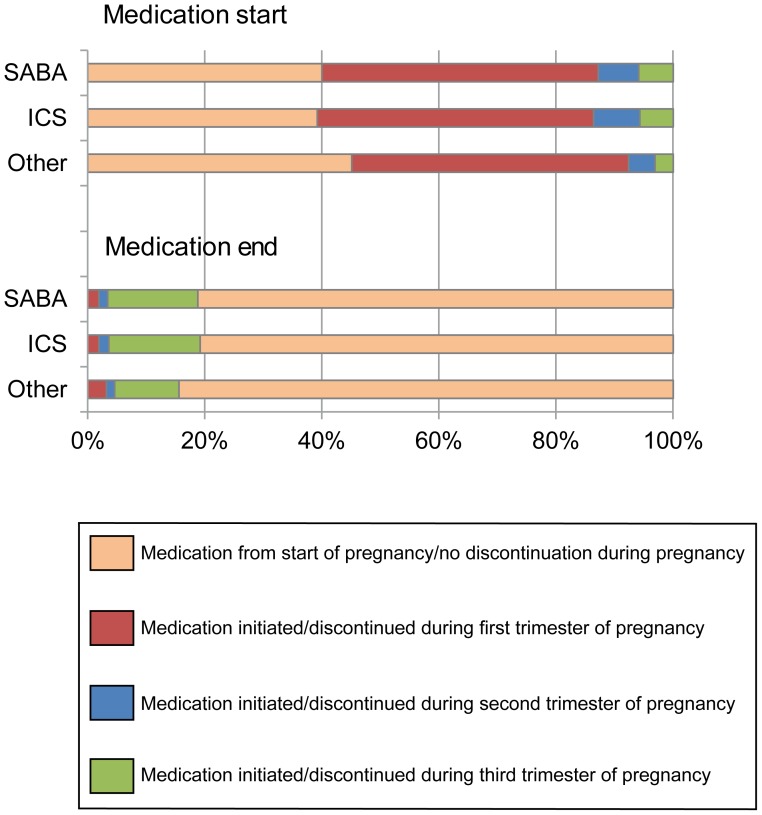
Asthma medication during trimesters. Starting and ending of treatment with asthma drugs (short-acting β2-agonists, SABA; inhaled corticosteroids, ICS and others (long-acting β2-agonists LABA and leukotriene receptor antagonist LTRA) in the Medical Birth Register.

In the Medical Birth Register, treatment with asthma drugs was most commonly already present at the start of pregnancy or initiated during the first trimester, Figure 2. Most women continued their medication throughout the whole pregnancy or ended medication during the third trimester. Short-acting β2-agonists (SABA) and inhaled corticosteroids (ICS) medication were slightly more often discontinued during pregnancy compared to other asthma medications (LABA and LTRA). We were not able to assess whether this group of women were at higher risk of pregnancy complications than those who did not discontinue. There was however an increased risk of adverse pregnancy outcomes in women with asthma medication noted in the Medical Birth Register, similar to the main results.

For analyses on the association between asthma severity (moderate/severe asthma with mild asthma as reference) and adverse outcomes, no increased risks for the described pregnancy outcomes were seen, Table 3. For the association between labour characteristics and birth outcomes there were significant findings for vaginal instrumental delivery, emergency CS, and the children born to women with moderate/severe asthma were significantly smaller compared to those born to women with mild asthma.

**Table 3:** Associations between moderate/severe asthma according to the Firoozi index the year before pregnancy and perinatal outcomes in a cohort of 284 214 pregnancies, mild asthma as reference.

	Cases in mild asthma group	Cases in moderate-severe asthma group	Moderate/severe asthma vs Mild asthma	Moderate/severe asthma vs Mild asthma	P-values adjusted
	n (%)	n (%)	Unadjusted OR (95% CI)	Adjusted* OR (95% CI)	
**Pregnancy characteristics**					
***Preeclampsia or eclampsia***	319 (3.6)	159 (3.9)	1.10 (0.89–1.35)	1.01 (0.81–1.26)	0.927
***Gestational diabetes***	188 (2.1)	85 (2.1)	0.99 (0.76–1.30)	0.97 (0.73–1.30)	0.850
***Haemorrhage during pregnancy***	54 (0.6)	27 (0.7)	1.10 (0.70–1.73)	1.13 (0.70–1.83)	0.612
***Premature contractions***	72 (0.8)	29 (0.7)	0.89 (0.56–1.41)	0.92 (0.59–1.43)	0.715
***Premature rupture of membranes***	167 (1.9)	88 (2.2)	1.16 (0.90–1.50)	1.20 (0.92–1.57)	0.182
***Placental abruption***	44 (0.5)	19 (0.5)	0.95 (0.53–1.71)	0.80 (0.46–1.40)	0.444
**Labour characteristics**					
***Labour dystocia***	1077 (12.0)	497 (12.2)	1.02 (0.91–1.14)	1.06 (0.94–1.19)	0.364
***Mode of delivery***					
*Vaginal, non-instrumental delivery*	6394 (71.3)	2767 (68.0)	Ref.	Ref.	
*Elective CS (before start of labour)*	732 (8.2)	358 (8.8)	1.13 (0.99–1.30)	0.99 (0.85–1.14)	0.861
*Vaginal instrumental delivery*	742 (8.3)	373 (9.2)	1.16 (1.01–1.34)	1.19 (1.03–1.37)	0.020
*Emergency CS prior to or after onset of labour*	1024 (11.4)	537 (13.2)	1.21 (1.08–1.36)	1.18 (1.04–1.33)	0.012
*Missing*	70 (0.8)	37 (0.9)			
***Haemorrhage after delivery***	499 (5.6)	222 (5.5)	0.98 (0.83–1.15)	1.03 (0.86–1.24)	0.728
**Birth outcome and post-partum**					
***Birth weight, grams***					
*≤1999*	123 (1.4)	56 (1.4)	1.07 (0.77–1.48)	0.99 (0.68–1.44)	0.964
*2000*–*2499*	184 (2.1)	113 (2.8)	1.44 (1.14–1.82)	1.45 (1.13–1.86)	0.004
*2500*–*2999*	966 (10.8)	539 (13.2)	1.31 (1.17–1.47)	1.39 (1.22–1.57)	<0.001
*3000*–*3499*	2907 (32.4)	1328 (32.6)	1.07 (0.99–1.16)	1.14 (1.04–1.25)	0.004
*≥3500*	4766 (53.2)	2031 (49.9)	Ref.	Ref.	
*Missing*	16 (0.2)	5 (0.1)			
***Gestational age, weeks***					
*≤31*	82 (0.9)	38 (0.9)	1.03 (0.69–1.54)	0.92 (0.59–1.42)	0.696
*32*–*34*	107 (1.2)	47 (1.2)	0.98 (0.68–1.40)	0.96 (0.66–1.39)	0.813
*35*–*36*	318 (3.5)	154 (3.8)	1.08 (0.88–1.32)	1.04 (0.84–1.28)	0.719
*37*–*38*	1802 (20.1)	863 (21.2)	1.07 (0.96–1.18)	1.05 (0.94–1.16)	0.399
*39*–*40*	4399 (49.1)	1978 (48.6)	Ref.	Ref.	
*≥41*	2254 (25.2)	992 (24.4)	0.98 (0.89–1.07)	0.99 (0.90–1.09)	0.824
*Missing*	0 (0.0)	0 (0.0)			
***Small for gestational age***					
*Yes*	204 (2.3)	153 (3.8)	1.68 (1.35–2.08)	1.71 (1.34–2.17)	<0.001
*Missing*	16 (0.2)	5 (0.1)			
***Large for gestational age***					
*Yes*	375 (4.2)	127 (3.1)	0.74 (0.60–0.91)	0.65 (0.52–0.81)	<0.001
*Missing*	16 (0.2)	5 (0.1)			
***Apgar at 5 minutes <7***	132 (1.5)	69 (1.7)	1.15 (0.86–1.54)	1.17 (0.86–1.59)	0.320
***Asphyxia or hypoxia***	110 (1.2)	53 (1.3)	1.06 (0.76–1.49)	1.12 (0.80–1.57)	0.495

Unadjusted and adjusted model (n = 249 006) estimated by multinomial logistic regression with OR and 95% CI. In unadjusted analyses n = 8962 were included in the mild asthma group and n = 4072 in the moderate/severe group. In the adjusted analyses n = 7996 were included in the mild and n = 3646 in the moderate/severe group.

*Adjusted for age, BMI, parity, smoking at antenatal care admission, country of birth, cohabitation/marital status and level of education.

In analyses of asthma control (uncontrolled versus controlled), for most outcomes we couldn't detect significant differences. However, pregnant women with uncontrolled asthma were at increased risk of instrumental vaginal delivery (OR 1.17 95% CI 1.03–1.34) compared to women with controlled asthma. An uncontrolled asthma also seemed to decrease the risk of premature contractions (OR 0.63, 95% CI 0.42–0.95) and low Apgar (OR 0.72 95% CI 0.52–0.99). There was significant effect modification (interaction) between asthma control and severity for premature rupture of the membranes (p = 0.007) and gestational age (p = 0.011). Uncontrolled asthma reduced the odds for premature rupture of the membranes for women with mild asthma (OR 0.65 95% CI 0.47–0.90), but not moderate/severe (OR 1.44 95% CI 0.88–2.35). For gestational age, there was a significantly increased risk of giving birth in week 37–38 if having uncontrolled asthma (OR 1.29 95% CI 1.07–1.56) among women with moderate/severe asthma but not among those with mild asthma, not shown in table.

## Discussion

In this population-based cohort study of 284 214 pregnancies, we found that maternal asthma is a risk factor for a number of adverse pregnancy and labour outcomes such as preeclampsia/eclampsia and caesarean section, and perinatal outcomes such as SGA. We also showed an increased risk of some adverse outcomes with increasing asthma severity the year prior to pregnancy, including instrumental delivery, emergency caesarean section, birth weight 2000–3499 grams and SGA. The adverse impact of asthma in pregnancy on most outcomes is striking. Although increased risks of haemorrhage during pregnancy, premature contractions, placental abruption and SGA have been observed in some previous studies [5], [6], [9], [12]–[14], [28]–[30], the novelty with our results is the consistency in risk increases for women with asthma before and during pregnancy across all pregnancy and labour characteristics, as well as most perinatal outcomes. This has not been previously shown and has important public health implications.

We observed higher rate of caesarean section in asthma patients which is in agreement with most previous studies [9], [10], [13], [31]. Higher rate of CS has also been observed in other chronic conditions [32]–[34] and it has been proposed that the higher rates of delivery interventions might be related to foetal stress associated with the underlying disease. However, we did not find an association between asthma and low Apgar score or child hypoxia or asphyxia in the registers. It is possible that women with chronic disease in general are more likely to undergo surgery for a medical reason or because of health care professionals' assumption that vaginal delivery is associated with more complications [35].

We did not identify asthma as a risk factor for preterm delivery <32 weeks which is consistent with one previous study [8]. There was however an increased risk of late preterm birth (GA 32–36 weeks). In the past, much focus has been on infant mortality and morbidity in the very or extremely premature children but it is now clear that even moderate or late prematurity are risk factors for the child [36].

Some speculate that increased risks of preterm delivery among women with asthma may be due to similarities between bronchial and uterine smooth muscle hyperresponsiveness [30]. The association between maternal asthma in pregnancy and adverse outcomes may be caused by periods of intermittent hypoxia during pregnancy affecting oxygenation of placental and cord blood which in turn has a negative influence on the foetus. Such an interpretation is supported by the finding of a correlation between low FEV_1_ during pregnancy and intrauterine growth retardation [37]. This hypothesis would also be supported if there were higher risk of pregnancy complications in women who discontinue their asthma medication during pregnancy. However we were not able to show this, perhaps due to the fact that in some women asthma improves during pregnancy [38]. There was an increased risk of adverse pregnancy outcomes such as placental abruption in women with asthma medication noted in the Medical Birth Register and hypoxia in the placenta could be a mechanism for abruption [39].

In separate analyses using asthma medication and diagnoses from the three separate registers, we observed increased risks of adverse outcomes similar to that of the overall analysis. This would suggest that asthma as such plays a key role in the adverse outcomes.

Women with uncontrolled asthma were at increased risk of instrumental vaginal delivery but also had decreased risk of premature contractions and low Apgar score and there was a slight interaction between asthma control and severity. These might be random findings or due to the overrepresented mild asthma group, but the lower risk of premature contractions might also be due to use of high doses of short acting beta agonists which are known to inhibit contractions of the uterus [40]. The findings should be further investigated in a clinical prospective cohort study on asthma severity, control and medication, where also discontinuation of medication can be further assessed.

The present study is the largest prospective to date with several strengths. Firstly, it is a population-based, longitudinal register based study in a unified health care environment with recording in medical registers of 280 000 singleton pregnancies in Sweden. In a cohort of the total population, we have the advantage that the results are representative and thus easily generalisable to the general population. Secondly, information on exposures and outcomes were prospectively collected, which precludes recall bias. Thirdly, the asthma diagnosis was established by applying predetermined asthma criteria to the National Patient Register (covers inpatient and outpatient visits but not visits to general practitioners), the Prescribed Drug Register (full coverage of all dispensed asthma medication); and the Swedish Medical Birth Register (covers approximately 99% of the pregnancies >22 weeks [23], [41]). In addition, the large sample gave us high statistical power.

There are also inherent limitations in register based studies. First, there may be residual confounding due to factors not recorded in the registers. We lack information on genetic factors, many pregnancy associated factors (e.g. maternal diet and passive smoking, physical activity or alcohol usage during pregnancy), and other factors related to both maternal asthma and pregnancy outcomes (e.g. health care utilisation patterns) [42]. Secondly, some posts in the Medical Birth Register are derived from automatic linking from other national registers whereas other variables such as asthma and medication rely on self-reporting to the midwife. Stephansson et al recently showed that the rate of reporting of medication in the Medical Birth Register is higher for more severe and chronic diseases. For asthma the agreement between the Prescribed Drug Register and the Medical Birth Register is around 58% [43]. We had however also access to maternal asthma diagnosis in the National Patient Register as well as medication in the Prescribed Drug Register, and detected higher estimates for the association between maternal asthma during pregnancy and outcomes in the National Patient Register and Prescribed Drug Register compared to the Medical Birth Register. Thirdly, asthma severity was assessed with an index developed in another country (Canada) and although Sweden and Canada are similar in many ways it's not certain the index validity is the same in Sweden. Our results indicate a need for increased awareness among midwives and obstetricians on the adverse effects of maternal asthma during pregnancy.

By increasing awareness of the population with maternal asthma before and during pregnancy and by doing so ensuring proper management, pregnancy and labour characteristics and perinatal outcomes would most likely improve. A study to test clinical guidelines for asthma in early pregnancy with a possibility of early asthma detection and asthma control intervention might be useful to women who are at risks of pregnancy complications, and a next step in further understanding the effects of the disease in pregnancy.

In conclusion, maternal asthma during pregnancy is associated with an increased risk of a number of serious pregnancy and labour complications and adverse perinatal outcomes. There is also an increased risk of a few adverse outcomes based on asthma severity and control. Greater awareness and improved asthma management would most likely improve outcomes.

## Supporting Information

Appendix S1 **Asthma severity according to Firoozi et al** [21] **.**
(DOCX)

## References

[1] Kwon HL, Belanger K, Bracken MB (2003) Asthma prevalence among pregnant and childbearing-aged women in the United States: estimates from national health surveys. Ann Epidemiol 13: 317–324. 12821270 10.1016/s1047-2797(03)00008-5

[2] Alexander S, Dodds L, Armson BA (1998) Perinatal outcomes in women with asthma during pregnancy. Obstet Gynecol 92: 435–440. 9721785 10.1016/s0029-7844(98)00191-4

[3] Murphy VE, Clifton VL, Gibson PG (2006) Asthma exacerbations during pregnancy: incidence and association with adverse pregnancy outcomes. Thorax 61: 169–176. 16443708 10.1136/thx.2005.049718 PMC2104591

[4] Schatz M, Dombrowski MP, Wise R, Thom EA, Landon M, et al (2003) Asthma morbidity during pregnancy can be predicted by severity classification. J Allergy Clin Immunol 112: 283–288. 12897733 10.1067/mai.2003.1516

[5] Wen SW, Demissie K, Liu S (2001) Adverse outcomes in pregnancies of asthmatic women: results from a Canadian population. Ann Epidemiol 11: 7–12. 11164114 10.1016/s1047-2797(00)00077-6

[6] Källén B, Rydhstroem H, Aberg A (2000) Asthma during pregnancy–a population based study. Eur J Epidemiol 16: 167–171. 10845267 10.1023/a:1007678404911

[7] Doucette JT, Bracken MB (1993) Possible role of asthma in the risk of preterm labor and delivery. Epidemiology 4: 143–150. 8452903 10.1097/00001648-199303000-00010

[8] Dombrowski MP, Schatz M, Wise R, Momirova V, Landon M, et al (2004) Asthma during pregnancy. Obstet Gynecol 103: 5–12. 14704237 10.1097/01.AOG.0000103994.75162.16

[9] Enriquez R, Griffin MR, Carroll KN, Wu P, Cooper WO, et al (2007) Effect of maternal asthma and asthma control on pregnancy and perinatal outcomes. J Allergy Clin Immunol 120: 625–630. 17658591 10.1016/j.jaci.2007.05.044

[10] Tata LJ, Lewis SA, McKeever TM, Smith CJ, Doyle P, et al (2007) A comprehensive analysis of adverse obstetric and pediatric complications in women with asthma. Am J Respir Crit Care Med 175: 991–997. 17272783 10.1164/rccm.200611-1641OC

[11] Källén B, Otterblad Olausson P (2007) Use of anti-asthmatic drugs during pregnancy. 1. Maternal characteristics, pregnancy and delivery complications. Eur J Clin Pharmacol 63: 363–373. 17265060 10.1007/s00228-006-0257-1

[12] Breton MC, Beauchesne MF, Lemiere C, Rey E, Forget A, et al (2009) Risk of perinatal mortality associated with asthma during pregnancy. Thorax 64: 101–106. 19008298 10.1136/thx.2008.102970

[13] Liu S, Wen SW, Demissie K, Marcoux S, Kramer MS (2001) Maternal asthma and pregnancy outcomes: a retrospective cohort study. Am J Obstet Gynecol 184: 90–96. 11174486 10.1067/mob.2001.108073

[14] Aly H, Nada A, Ahmad T, Mohamed M, Massaro AN, et al (2011) Maternal asthma, race and low birth weight deliveries. Early Hum Dev 87: 457–460. 21511412 10.1016/j.earlhumdev.2011.03.007

[15] Källén B, Otterblad Olausson P (2007) Use of anti-asthmatic drugs during pregnancy. 2. Infant characteristics excluding congenital malformations. Eur J Clin Pharmacol 63: 375–381. 17265059 10.1007/s00228-006-0258-0

[16] Clifton VL, Engel P, Smith R, Gibson P, Brinsmead M, et al (2009) Maternal and neonatal outcomes of pregnancies complicated by asthma in an Australian population. Aust N Z J Obstet Gynaecol Australia. pp 619–626. 20070710 10.1111/j.1479-828X.2009.01077.x

[17] (GINA) GIfA (2012) Global Strategy for Asthma Management and Prevention.

[18] Namazy JA, Murphy VE, Powell H, Gibson PG, Chambers C, et al (2013) Effects of asthma severity, exacerbations and oral corticosteroids on perinatal outcomes. Eur Respir J 41: 1082–1090. 22903964 10.1183/09031936.00195111

[19] Murphy VE, Namazy JA, Powell H, Schatz M, Chambers C, et al (2011) A meta-analysis of adverse perinatal outcomes in women with asthma. BJOG 118: 1314–1323. 21749633 10.1111/j.1471-0528.2011.03055.x

[20] Ungar WJ, Chapman KR, Santos MT (2002) Assessment of a medication-based asthma index for population research. Am J Respir Crit Care Med 165: 190–194. 11790653 10.1164/ajrccm.165.2.2102012

[21] Firoozi F, Lemiere C, Beauchesne MF, Forget A, Blais L (2007) Development and validation of database indexes of asthma severity and control. Thorax. England pp. 581–587. 10.1136/thx.2006.061572 PMC2117251 17287299

[22] Ludvigsson JF, Otterblad-Olausson P, Pettersson BU, Ekbom A (2009) The Swedish personal identity number: possibilities and pitfalls in healthcare and medical research. Eur J Epidemiol 24: 659–667. 19504049 10.1007/s10654-009-9350-y PMC2773709

[23] Swedish National Board of Health and Welfare CfE (2003) The Swedish Medical Birth Register - A summary of content and quality.

[24] Wettermark B, Harnmar N, MichaelFored C, Leimanis A, Olausson PO, et al (2007) The new Swedish Prescribed Drug Register - Opportunities for pharmacoepidemiological research and experience from the first six months. Pharmacoepidemiology and Drug Safety 16: 726–735. 16897791 10.1002/pds.1294

[25] Ortqvist AK, Lundholm C, Wettermark B, Ludvigsson JF, Ye W, et al (2013) Validation of asthma and eczema in population-based Swedish drug and patient registers. Pharmacoepidemiol Drug Saf 22: 850–860. 23754713 10.1002/pds.3465

[26] Ludvigsson JF, Andersson E, Ekbom A, Feychting M, Kim JL, et al (2011) External review and validation of the Swedish national inpatient register. Bmc Public Health 11: 16. 21658213 10.1186/1471-2458-11-450 PMC3142234

[27] Marsal K, Persson PH, Larsen T, Lilja H, Selbing A, et al (1996) Intrauterine growth curves based on ultrasonically estimated foetal weights. Acta Paediatr 85: 843–848. 8819552 10.1111/j.1651-2227.1996.tb14164.x

[28] Bahna SL, Bjerkedal T (1972) The course and outcome of pregnancy in women with bronchial asthma. Acta Allergol 27: 397–406. 4679026 10.1111/j.1398-9995.1972.tb01439.x

[29] Sorensen TK, Dempsey JC, Xiao R, Frederick IO, Luthy DA, et al (2003) Maternal asthma and risk of preterm delivery. Ann Epidemiol 13: 267–272. 12684193 10.1016/s1047-2797(02)00413-1

[30] Kramer MS, Coates AL, Michoud MC, Dagenais S, Moshonas D, et al (1995) Maternal asthma and idiopathic preterm labor. Am J Epidemiol 142: 1078–1088. 7485053 10.1093/oxfordjournals.aje.a117561

[31] Perlow JH, Montgomery D, Morgan MA, Towers CV, Porto M (1992) Severity of asthma and perinatal outcome. Am J Obstet Gynecol 167: 963–967. 1415433 10.1016/s0002-9378(12)80020-2

[32] Stephansson O, Larsson H, Pedersen L, Kieler H, Granath F, et al (2010) Congenital abnormalities and other birth outcomes in children born to women with ulcerative colitis in Denmark and Sweden. Inflamm Bowel Dis 10.1002/ibd.21369 20564537

[33] Stephansson O, Larsson H, Pedersen L, Kieler H, Granath F, et al (2010) Crohn's disease is a risk factor for preterm birth. Clin Gastroenterol Hepatol United States: 2010 AGA Institute. Published by Elsevier Inc pp. 509–515. 10.1016/j.cgh.2010.02.014 20202483

[34] Borthen I, Eide MG, Daltveit AK, Gilhus NE (2011) Obstetric outcome in women with epilepsy: a hospital-based, retrospective study. BJOG 118: 956–965. 21557799 10.1111/j.1471-0528.2011.03004.x

[35] Linton A, Peterson MR (2004) Effect of preexisting chronic disease on primary cesarean delivery rates by race for births in U.S. military hospitals, 1999-2002. Birth 31: 165–175. 15330878 10.1111/j.0730-7659.2004.00301.x

[36] Raju TN (2006) Epidemiology of late preterm (near-term) births. Clin Perinatol 33: 751–763 abstract vii. 17148002 10.1016/j.clp.2006.09.009

[37] Schatz M, Zeiger RS, Hoffman CP (1990) Intrauterine growth is related to gestational pulmonary function in pregnant asthmatic women. Kaiser-Permanente Asthma and Pregnancy Study Group. Chest 98: 389–392. 2376171 10.1378/chest.98.2.389

[38] Schatz M, Harden K, Forsythe A, Chilingar L, Hoffman C, et al (1988) The course of asthma during pregnancy, post partum, and with successive pregnancies: a prospective analysis. J Allergy Clin Immunol 81: 509–517. 3346481

[39] Getahun D, Ananth CV, Peltier MR, Smulian JC, Vintzileos AM (2006) Acute and chronic respiratory diseases in pregnancy: associations with placental abruption. Am J Obstet Gynecol 195: 1180–1184. 17000252 10.1016/j.ajog.2006.07.027

[40] Simhan HN, Caritis SN (2007) Prevention of preterm delivery. N Engl J Med 357: 477–487. 17671256 10.1056/NEJMra050435

[41] Axelsson O (2003) The Swedish medical birth register. Acta Obstet Gynecol Scand 82: 491–492. 12780418 10.1034/j.1600-0412.2003.00172.x

[42] Manderbacka K, Keskimaki I, Reunanen A, Klaukka T (2008) Equity in the use of antithrombotic drugs, beta-blockers and statins among Finnish coronary patients. Int J Equity Health 7: 16. 18590524 10.1186/1475-9276-7-16 PMC2459171

[43] Stephansson O, Granath F, Svensson T, Haglund B, Ekbom A, et al (2011) Drug use during pregnancy in Sweden - assessed by the Prescribed Drug Register and the Medical Birth Register. Clin Epidemiol 3: 43–50. 21386973 10.2147/CLEP.S16305 PMC3046184

