# Supplementary material for: Asthma during Pregnancy in a Population-Based Study - Pregnancy Complications and Adverse Perinatal Outcomes
Source: PLoS One. 2014 Aug 20;9(8):e104755. doi: 10.1371/journal.pone.0104755 (PMC4139314; doi:10.1371/journal.pone.0104755)
Supplement: Appendix S1 — Asthma severity according to Firoozi et al [21] . (DOCX) [file pone.0104755.s001.docx]

**Appendix S1.**

Asthma severity according to Firoozi et al [21].

| **Asthma severity and control** |  | **Inhaled corticosteroids (ICS), daily dose, µg** (daily dose of ICS in beclomethasone-chlorofluorocarbin equivalent over a 12 month period) | **Other controller therapy** (at least two prescriptions or long-acting beta agonsits (LABA), theophylline or leukotriene receptor antagonists (LTRA) dispensed over a 12 months period) | **Short acting beta agonists (SABA) doses per week** (average doses inhaled SABA per week calculated over a 12 month period) | **Marker of moderate to severe exacerbations** (an emergency department visit for asthma, a hospital admission for asthma or a filled prescription of an oral corticosteroid over a 12 month period) |
| --- | --- | --- | --- | --- | --- |
| Mild asthma |  |  |  |  |  |
|  | Controlled | 0-500 | No | 0-3 | No |
|  |  | 0-250 | Yes | 0-3 | No |
|  | Uncontrolled | 0-250 | Yes | 0-3 | Yes |
|  |  | 0-500 | No | 0-3 | Yes |
|  |  | 0-250 | Yes | 4-10 | No |
|  |  | 0-500 | No | 4-10 | No |
| Moderate asthma |  |  |  |  |  |
|  | Controlled | 251-500 | Yes | 0-10 | No |
|  |  | 501-1000 | Yes/No | 0-10 | No |
|  |  | >1000 | Yes/No | 0-3 | No |
|  | Uncontrolled | 0-250 | Yes | 4-10 | Yes |
|  |  | 0-500 | No | 4-10 | Yes |
|  |  | 0-250 | Yes | >10 | No |
|  |  | 0-500 | No | >10 | No |
|  |  | 251-500 | Yes | >10 | No |
|  |  | 251-500 | Yes | 0-10 | Yes |
|  |  | 501-1000 | Yes/No | >10 | No |
|  |  | 501-1000 | Yes/No | 0-10 | Yes |
| Severe asthma |  |  |  |  |  |
|  | Controlled | >1000 | Yes/No | 4-10 | No |
|  | Uncontrolled | 0-1000 | Yes/No | >10 | Yes |
|  |  | >1000 | Yes/No | 0-10 | Yes |
|  |  | >1000 | Yes/No | >10 | Yes/No |
